# Actin and myosin II modulate differentiation of pluripotent stem cells

**DOI:** 10.1371/journal.pone.0195588

**Published:** 2018-04-17

**Authors:** Liana C. Boraas, Emma T. Pineda, Tabassum Ahsan

**Affiliations:** 1 Department of Biomedical Engineering, Tulane University, New Orleans, Louisiana, United States of America; 2 RoosterBio Inc., Frederick, Maryland, United States of America; University of Kansas Medical Center, UNITED STATES

## Abstract

Use of stem cell-based therapies in tissue engineering and regenerative medicine is hindered by efficient means of directed differentiation. For pluripotent stem cells, an initial critical differentiation event is specification to one of three germ lineages: endoderm, mesoderm, and ectoderm. Differentiation is known to be regulated by numerous extracellular and intracellular factors, but the role of the cytoskeleton during specification, or early differentiation, is still unknown. In these studies, we used agonists and antagonists to modulate actin polymerization and the actin-myosin molecular motor during spontaneous differentiation of embryonic stem cells in embryoid bodies. We found that inhibiting either actin polymerization or actin-myosin interactions led to a decrease in differentiation to the mesodermal lineage and an increase in differentiation to the endodermal lineage. Thus, targeting processes that regulate cytoskeletal tension may be effective in enhancing or inhibiting differentiation towards cells of the endodermal or mesodermal lineages, which include hepatocytes, islets, cardiomyocytes, endothelial cells, and osteocytes. Therefore, these fundamental findings demonstrate that modulation of the cytoskeleton may be useful in production for a range of cell-based therapies, including for liver, pancreatic, cardiac, vascular, and orthopedic applications.

## Introduction

Understanding directed differentiation is critical for the proper and efficient development of stem cell-based tissue engineering therapies. The emergence of personalized medicine, based on induced pluripotent stem cells, has increased the importance of determining the regulators of the early differentiation events that occur with loss of pluripotency. Specification to one of the three germ lineages (ectoderm, endoderm, or mesoderm) is the critical first step in directing differentiation to downstream functional phenotypes. Therefore, identifying means to regulate early specification can be important in effectively promoting downstream differentiation to therapeutically-relevant phenotypes.

Stem cell differentiation is a complex process regulated both spatially and temporally by numerous extracellular and intracellular factors. One regulator of differentiation in more developed multipotent adult stem cells is the cytoskeleton, a complex network of structural filaments. In mesenchymal stem cells (MSCs) the use of small molecule antagonists to disrupt actin polymerization [1] or actin-myosin interactions [2] resulted in an increase in adipogenic differentiation and a decrease in osteogenic differentiation. During the differentiation process, however, the timing of specific cues can have alternate effects dependent on cell state. To generate cardiac progenitors from pluripotent stem cells, for example, the Wnt signaling pathway must be activated early but inhibited later [3]. Application of cyclic compression to multipotent MSCs promoted chondrogenic differentiation while the same mechanical cue inhibited chondrogenic differentiation in more naïve cells derived from pluripotent stem cells [4]. While actin polymerization and actin-myosin interactions have been shown to regulate multipotent stem cell differentiation, it is unknown if or how those cytoskeletal processes regulate differentiation of naïve pluripotent stem cells.

Previous studies in pluripotent stem cells have investigated the role of actin polymerization or actin-myosin interactions on self-renewal and pluripotency under expansion conditions meant to inhibit differentiation. In those studies, inhibition of the Rho/Rock pathway, which regulates actin-myosin contractility or inhibition of actin-myosin interactions, directly increased cell survival and cloning efficiency [5,6], as well as increased expression of pluripotency markers [5]. Inhibition of actin polymerization also increased pluripotent stem cell survival but did not increase cloning efficiency [6]. Despite this extensive evaluation in the pluripotent state, the role of actin polymerization and actin-myosin interactions has yet to be determined in pluripotent stem cells during early germ lineage specification and downstream differentiation.

Incomplete reprogramming and uncharacterized residual properties from the parental phenotype currently complicate the use of induced pluripotent stem cell populations in fundamental studies of early differentiation [7,8,9]. The role of the cytoskeleton in germ lineage specification can therefore be more readily elucidated in a well characterized embryonic stem cell population. Using embryonic stem cells (ESCs) that were more homogenous than an induced population, we found that pluripotent stem cells have minimal structural organization and low levels of cytoskeletal expression [10]. Expression of actin and intermediate filaments then increased with differentiation of these ESCs under multiple 2D and 3D culture conditions [11]. To determine if the cytoskeleton regulates differentiation to the germ lineages, however, requires selection of a suitable *in vitro* model. Use of the embryoid body model allows for study of spontaneous germ lineage differentiation in a 3D *in vitro* configuration that self-assembles similar to the *in vivo* process [12], avoiding additional artifacts associated with 2D culture such as restriction to monolayer growth and adherence to a protein-coated stiff surface.

**Fig 1:**
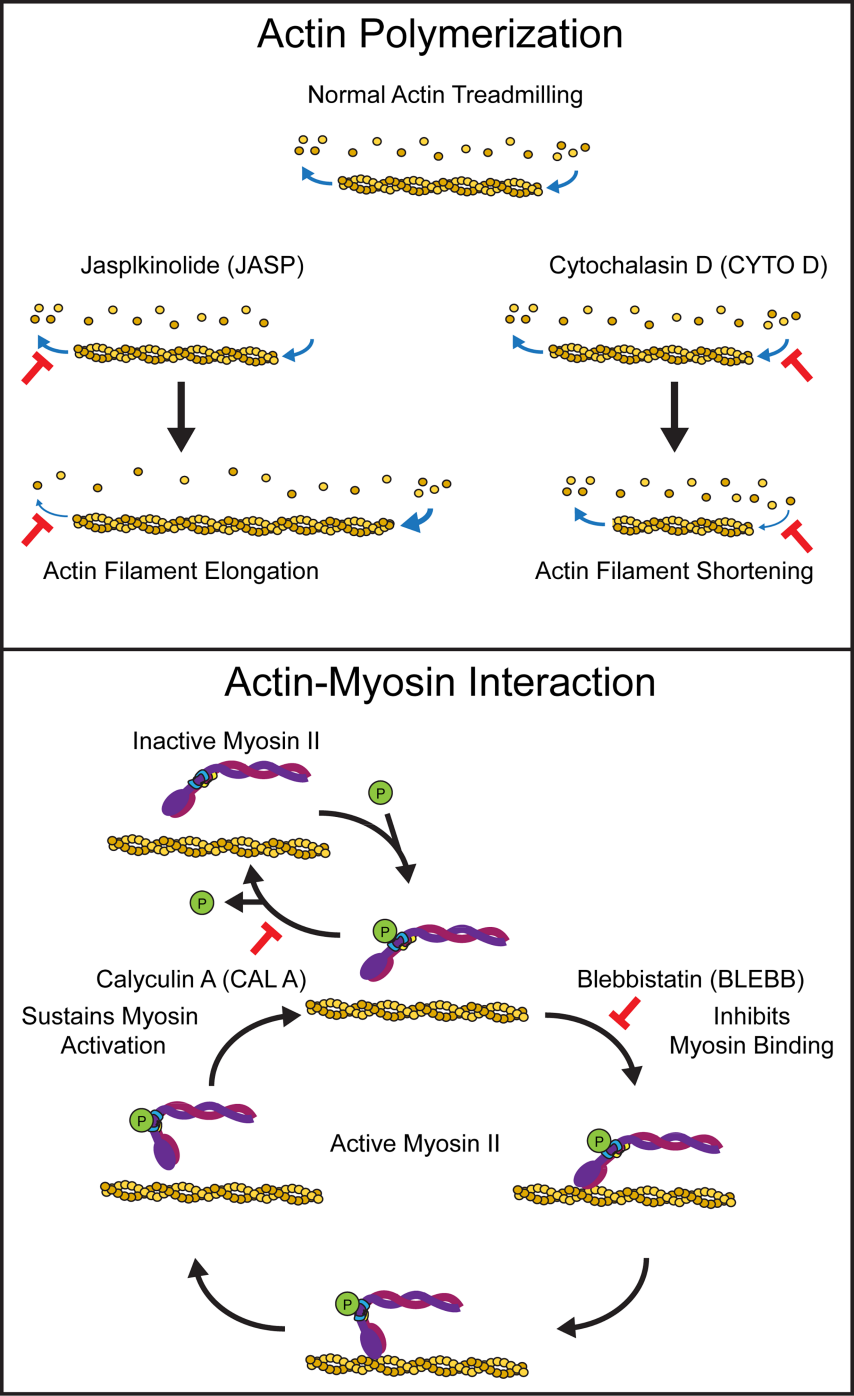
Perturbation of actin polymerization or actin-myosin interactions. Normal actin polymerization is perturbed in cells treated with either the agonist jasplkinolide (JASP), which increases actin filament length by preventing depolymerization, or the antagonist cytochalasin D (CYTO D), which results in actin filament shortening by preventing polymerization. Actin-myosin interaction is perturbed in cells treated with either the agonist calyculin A (CAL A), which increases actin-myosin interactions by inhibiting the deactivation of myosin, or the antagonist blebbistatin (BLEBB) which decreases actin-myosin interactions by inhibiting binding of myosin to actin.

The focus of these studies was to modulate the cytoskeleton and evaluate its role during early differentiation. In particular, our objective was to determine the effects of agonists and antagonists of actin polymerization and actin-myosin interactions (Fig 1) on germ lineage specification during spontaneous ESC differentiation as embryoid bodies. Overall, we found that actin polymerization and actin-myosin interactions can serve as targets to modulate differentiation to mesodermal and endodermal phenotypes.

## Materials and methods

### Embryonic stem cell culture

Mouse D3 embryonic stem cells (ESCs; ATCC™, Manassas, VA) were cultured as described previously [13]. Briefly, cells were cultured on gelatin-coated tissue culture plastic with medium that consisted of Dulbecco’s Modification of Eagles Medium, 15% ESC-qualified fetal bovine serum (Invitrogen, Carlsbad, CA), 2 mM L-glutamine, 0.1 mM non-essential amino acids, antibiotics, and 1000 U/ml leukemia inhibitory factor (LIF; EMD Millipore, Temecula, CA).

### Differentiation conditions

Embryoid bodies (EBs) were generated by suspending ESCs (0.5x10^6^ cells) in 10 mL of medium without LIF in non-coated petri dishes. Samples were maintained at 40 RPM on a rotary shaker to prevent aggregation of EBs. After the second day, culture medium and dishes were changed daily by gravity separation. In experimental samples the medium was supplemented with one or more of the following small molecules: jasplakinolide (JASP; 50 nM; Millipore), cytochalasin D (CYTO D; 0.2 μM; Sigma Aldrich, St. Louis, MO), calyculin A (CAL A; 1 nM; Santa Cruz, Dallas Texas), or blebbistatin (BLEBB; 24 μM in 3D; Sigma Aldrich). Control samples for each group were instead supplemented with a matched volume of solvent (final concentration of <0.15% DMSO). Compared to controls, treatment with these small molecules did not notably increase cell death (S1 Fig). Treatment with supplemented medium was applied for 3 days (Days 4–7) after which samples were assessed for morphology or differentiation.

### Morphological assessment

Scanning electron microscopy (SEM) was used to determine the morphology of the EBs and the differentiating cells within. Both treated and untreated samples were fixed in 2.5% glutaraldehyde and a 1% osmium tetroxide / 0.1M sodium cacodylate solution (Sigma Aldrich®). EBs were then fractured, dehydrated, and sputter-coated with gold or carbon. Images were taken on a Hitachi 4800 system.

### Gene expression

Samples were evaluated for gene expression as described previously [13]. For each sample, 1 μg of RNA was isolated, converted into cDNA, and analyzed using standard real-time PCR with SYBR® Green on a StepOnePlus™ PCR System (Applied Biosystems, Foster City, CA). Primers were designed to assess mesodermal differentiation (mesenchyme homeobox 1: *Meox1*; paired box gene 2: *Pax2*; vascular endothelial growth factor receptor 2: *Flk1*) and endodermal differentiation (SRY-Related HMG-Box Transcription Factor: *Sox17*; Forkhead Box A2: *FoxA2*; alpha-fetoprotein: *Afp*). Gene expression levels were determined using standard curves and reported normalized to glyceraldehyde-3-phosphate dehydrogenase (*Gapdh*). Results are presented normalized to solvent matched controls.

### Protein expression

Protein expression was determined using standard protocols of immunohistochemistry. Both treated and control EB samples were fixed in 4% formaldehyde, paraffin embedded, and sectioned (6 μm thick) onto slides. Slide sections were then deparaffinized, heat-denatured, blocked for non-specific binding with serum, and then stained with primary and secondary antibodies, as well as HOECHST 33258 as a nuclear counterstain. Primary antibodies were against FLK1 (Santa Cruz) or AFP (Santa Cruz) and secondary antibodies were conjugated with AF488 or AF546 (Molecular Probes, Eugene, OR). Images were then taken with either an Olympus® fluorescent microscope or Nikon A1 Confocal microscope using identical voltage settings and conditions to image a particular protein in all samples.

### Statistical analysis

Data are presented as mean ± standard error of the mean. Comparisons between treated samples and their matched controls were analyzed using a student’s t-test for 3–4 replicates from each independent experimental trial, with multiple trials for the single treatment groups. P-values <0.05 were considered significant.

## Results and discussion

### Experimental design

**Fig 2:**
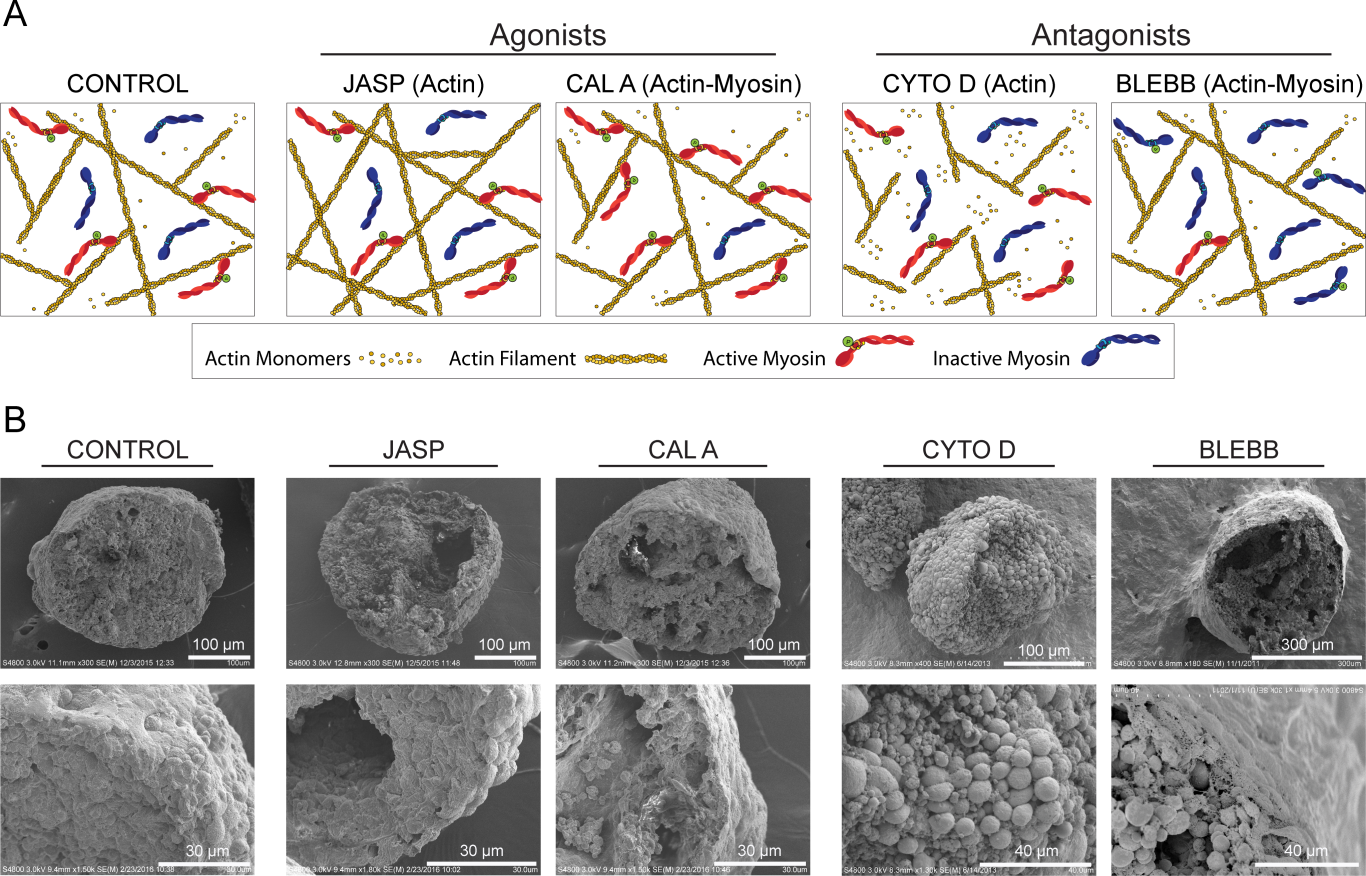
Altered actin polymerization or actin-myosin interactions disrupt EB morphology. (A) Diagram illustrating the relative state of actin and myosin II filaments in CONTROL, JASP, CAL A, CYTO D, or BLEBB treated cells (elements not to scale). (B) SEM images were taken at both low and high magnifications of fracture EBs. Length of scale bars is indicated in each image.

**Fig 3:**
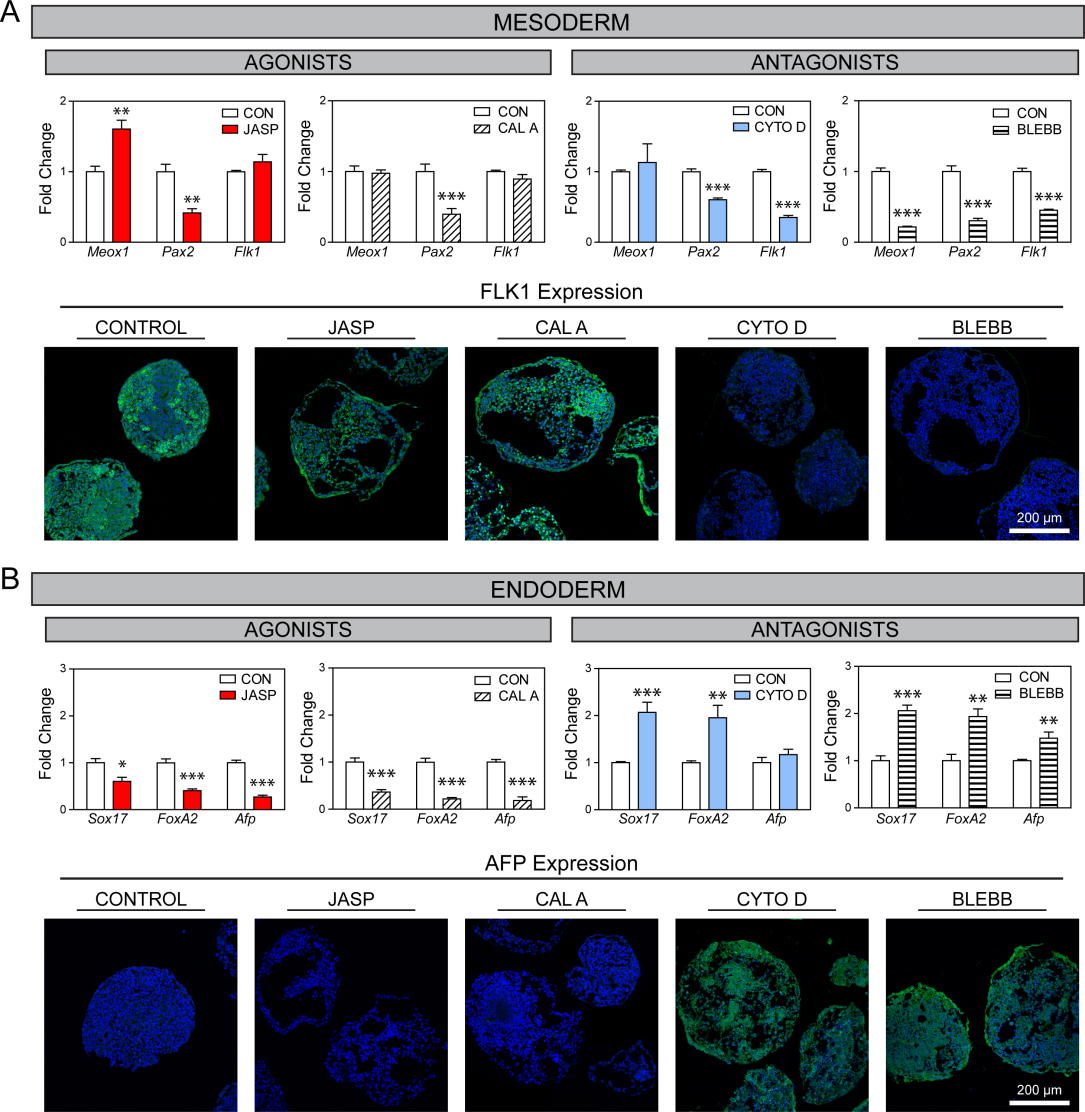
Perturbation of actin polymerization or actin-myosin interactions alter differentiation in EBs. Markers of mesodermal (A) and endodermal (B) differentiation in treated EBs. Gene results are shown normalized to solvent controls (mean ± SEM; n = 7–8). Asterisks indicate significance between treatment groups and matched controls (*p<0.05, **p<0.01, ***p<0.001). Immunohistochemical analysis of FLK1 or AFP (protein expression in green) with a nuclear counterstain (blue) in control and treated EBs. All images were taken at the same voltage settings and magnification, where the scale bar represents 200 μm.

**Fig 4:**
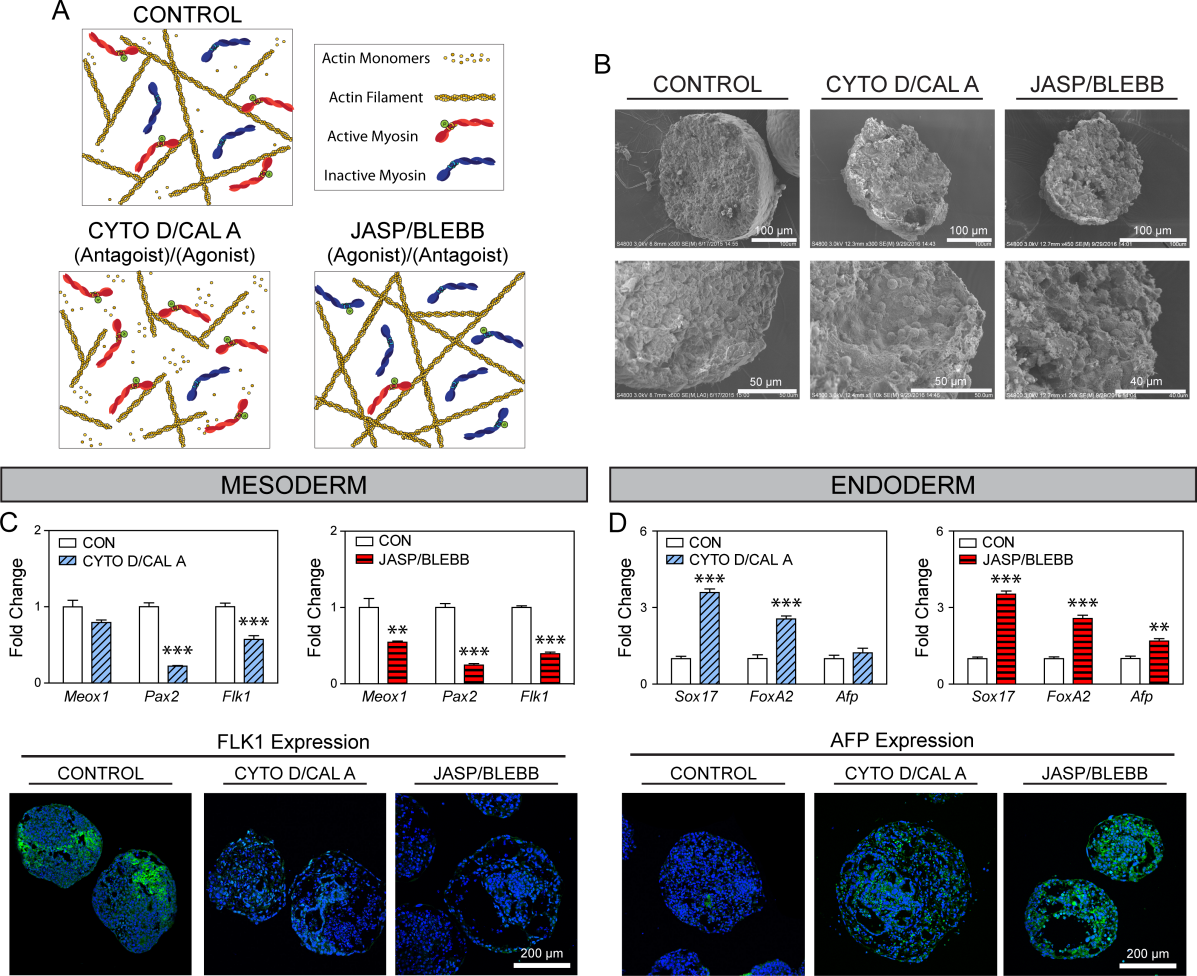
Effects of combined treatments on differentiation are dominated by antagonist. (A) Diagram illustrating the relative state of actin and myosin II filaments in CONTROL, CYTO D/CAL A, or JASP/BLEBB treated cells (elements not to scale). (B) SEM images were taken at both low and high magnifications of fracture EBs. Length of scale bars is indicated in each image. Markers of mesodermal (C) and endodermal (D) differentiation in treated EBs. Gene results are shown normalized to solvent controls (mean ± SEM; n = 4). Asterisks indicate significance between treatment groups and matched controls (**p<0.01, ***p<0.001). Immunohistochemical analysis of FLK1 or AFP (protein expression in green) with a nuclear counterstain (blue) in control and treated EBs. All images were taken at the same magnification and the scale bar represents 200 μm.

These studies utilized reagents to perturb the kinetics of actin polymerization and actin-myosin interactions during embryonic stem cell differentiation in embryoid bodies (EBs). Spontaneously differentiating EBs were treated from Day 4 to 7 (and assessed at Day 7) with reagents known to be agonists and antagonists of these cytoskeletal processes (Figs 1 and 2A). Actin filament formation was perturbed with jasplakinolide (JASP) to prevent depolymerization [14] or cytochalasin D (CYTO D) to inhibit polymerization [15], resulting in extended or shortened actin filaments, respectively. The actin-myosin molecular motor was modulated with calyculin A (CAL A) to inhibit deactivation of myosin [16] or blebbistatin (BLEBB) to inhibit binding of myosin to actin [17], resulting in increased and decreased actin-myosin interactions, respectively. Effects on morphology and differentiation were evaluated after treatment with either a single reagent (Figs 2 and 3) or a combination (Fig 4). Evaluation of specification was limited to the mesodermal and endodermal lineages as few ectodermal phenotypes emerge in this culture model without specific cytokine supplementation at these time points [18].

### Perturbation of actin polymerization or actin-myosin interactions altered differentiation

SEM analysis of solvent matched control samples at Day 7 have a smooth outer surface and few cavitations in the interior (Fig 2B). Treatment during culture with agonists, which elongate actin filaments (JASP) or increase actin-myosin interactions (CAL A), resulted in EBs with similar gross morphology but with an increase in cavitation. Treatment with the antagonist BLEBB also increased the number of cavitations observed. However, the primary effect of treatment with antagonists, which shorten actin filaments (CYTO D) or decrease actin-myosin interactions (BLEBB), was in altering the shape of individual cells with rounded cells visible throughout the interior of the EBs. Furthermore in the CYTO D treated samples, the change in cell shape in the outermost layer of cells led to a more rippled EB surface. These observed changes in morphology in treated samples are likely due to global changes in cytoskeletal structure within individual cells as the cytoskeleton is a known regulator of cell shape. Some morphological changes may also be due to perturbation of cytoskeletal connections to transmembrane complexes, altering cell-cell or cell-matrix interactions, as suggested by an altered distribution of E-cadherin positive cells within the EBs (S2 Fig).

Mesodermal differentiation was evaluated with gene expression of *Meox1*, *Pax2*, and *Flk1* for the paraxial, intermediate, and lateral mesodermal plates, respectively, in differentiating EBs (Fig 3A). Treatment with JASP or CAL A (agonists) had differential effects on each mesodermal marker: *Pax2* was significantly downregulated compared to control samples in both groups, *Meox1* was only upregulated with JASP treatment, and *Flk1* was unaffected in either group. Treatment with antagonists for actin polymerization (CYTO D) and actin-myosin interactions (BLEBB), however, had a more consistent effect across all markers. With the exception of *Meox1* in CYTO D treated samples, expression of all three mesodermal markers was significantly (p<0.001) downregulated by >40% compared to control samples. Immunohistochemical analysis for FLK1 protein corroborated the changes in gene expression. Control samples showed distinct areas of positive expression of FLK1 throughout the EBs. The agonists also had extensive positive expression with no notable difference compared to controls. Conversely, samples treated with the antagonists, and imaged under identical conditions as the other samples, had such low levels of expression that there was no observable immunostaining for FLK1 protein.

Endodermal differentiation was evaluated with gene expression of *Sox17*, *FoxA2*, and *Afp* (Fig 3B). Treatment with JASP and CAL A agonists resulted in a significant (p<0.05) downregulation by 40%-86% in all three endodermal markers compared to control samples. In a consistent manner, treatment with antagonists resulted in a statistically significant (p<0.01) increase in expression of *Sox17* and *FoxA2* by approximately 2-fold, as well as *Afp* (1.5-fold; BLEBB group only). Evaluation of AFP protein after spontaneous differentiation in the control group, as well as treatment with the cytoskeletal agonists, revealed little observable expression. Treatment with the antagonists to actin polymerization and actin-myosin interactions, however, promoted extensive expression of AFP protein throughout the EBs.

In spontaneously differentiating EBs, the use of cytoskeletal agonists and antagonists for actin polymerization and actin-myosin interactions did not maintain pluripotency (S3 Fig) but did affect both differentiation and morphology. Modulation of either cytoskeletal process led to similar findings. Inhibition of actin polymerization or actin-myosin interactions decreased differentiation towards the mesodermal phenotype, while promotion of these processes had little effect. The consistent decrease in mesodermal differentiation due to the CYTO D and BLEBB antagonists indicate that actin polymerization and actin-myosin interactions play a role in mesodermal differentiation. The lack of any observed increase in EBs treated with agonists, however, is likely due to the high baseline level of mesodermal differentiation. This is consistent with our own studies [11,19], as well as those by others [18], that have found that without treatment this model of spontaneously differentiating EBs has robust differentiation towards the mesodermal phenotype.

Protein expression of endodermal AFP remains low in control EBs even after seven days of differentiation. Samples that were treated with the cytoskeletal agonists also had no observable immunostaining for the AFP protein, as well as decreased gene expression of all evaluated endodermal markers. Treatment with the antagonists, however, not only had overall increased endodermal gene expression but also a distinct and marked increase in AFP protein expression. Therefore, modulation of actin polymerization and actin-myosin interactions can be used to both increase and decrease endodermal differentiation in EBs. Given this bidirectional sensitivity, endodermal differentiation may be a sensitive gauge of cytoskeletal perturbation in this culture model.

The loss of pluripotency in ESCs is associated with differentiation towards the three germ lineages: endoderm, mesoderm, and ectoderm. During spontaneous differentiation in EBs, endodermal and mesodermal phenotypes are readily detected [18]. In the absence of any lineage-specific supplemental cytokines at these time points, however, little to no differentiation is noted towards the ectodermal lineage [18]. Thus in this system with differentiation primarily to two lineages, it is rationale that the same treatment conditions led to observations at the population level of decreased mesoderm and increased endoderm. It cannot be determined from these studies, however, whether the cytoskeletal processes directly regulate endodermal differentiation, mesodermal differentiation, or both. Furthermore, modulation of the cytoskeleton, particularly in the case of the antagonists CYTO D [20] and BLEBB [21], has been previously established to alter cell shape in a variety of cell types [22]. In the application of a single reagent as above, it was therefore not possible to determine the independent effects of the observed changes in cell shape from changes in cytoskeletal processes. In the subsequent studies a combination of an agonist and antagonist was useful in starting to separate these effects.

### Differentiation in combinatorial perturbations were similar to antagonist alone

In these studies (Fig 4A), treatments were a combination of either CYTO D/CAL A (to decrease actin polymerization with an increase in levels of actin-myosin interactions) or JASP/BLEBB (to increase actin polymerization with a decrease in actin-myosin interactions). While individual treatments of JASP or BLEBB resulted in large cavitations (Fig 2B), the reagents in combination created cavities of a smaller size. CAL A treatment alone (the agonist of actin-myosin interactions) also induced small cavities (Fig 2B), which persisted even in the presence of the CYTO D (Fig 4B), the antagonist to actin polymerization. While rounded cells were pervasive in samples treated with either antagonist alone, few were visible in these samples treated with a combination of an antagonist with an agonist. The limited cell rounding or smaller cavitation size could be due to a counterbalancing effect of the agonist and antagonists actin on the actin-myosin network.

Differentiation patterns for the combination treatments mimicked those of the antagonist reagent alone. CYTO D/CAL A and JASP/BLEBB treatment groups resulted in a significant (p<0.01) downregulation of the mesodermal markers *Pax2* (by ~75% for both) and *Flk1* (by 42 or 61%, respectively), as well as *Meox1* (by 45%) in the JASP/BLEBB group (Fig 4C). Immunostaining of samples from both combination treatment groups also showed markedly lower levels of FLK1 protein compared to control samples. As seen with mesodermal differentiation, the endodermal effect of the combination treatments were similar to the effect of the antagonist alone. CYTO D/CAL A and JASP/BLEBB treated samples had a similar significant (p<0.001) increase in *Sox17* (by ~3.5-fold) and *FoxA2* (by ~2.5-fold), as well an increase in *Afp* for the JASP/BLEBB group (by 1.7-fold, p<0.01). Protein expression of AFP was extremely low in EBs of control samples. In the experimental groups, however, the combination treatments led to higher expression, where CYTO D/CAL A samples had an intense speckled pattern while the JASP/BLEBB EBs had a more consistent expression throughout the EBs.

Changes in cytoskeletal processes, particularly those related to actin polymerization [20] and actin-myosin interactions [21], have been shown to induce changes in the shapes of cells. Photolithography studies to control cell shape have also been shown to regulate differentiation [23,24], but changes in cytoskeletal state were also involved [25]. Similar studies that controlled for both cell shape and cytoskeletal processes directly also showed that actin-myosin interactions play a role in adult stem cell differentiation [1]. Also in the first studies presented here, the samples that were treated with single antagonists had individual cells with a more rounded morphology. In both those findings, therefore, it could not be determined if the effects on differentiation were due to the direct modulation of the cytoskeletal processes or its downstream effects on morphology. In our samples treated with a combination of an agonist and antagonists, however, there were no noticeable changes in cell morphology yet the effects on differentiation were similar to the antagonist only groups. While it is not known if the same mechanisms regulate differentiation for the different treatment groups, in the case of the combined treatments the effects of the cytoskeletal processes on differentiation seem to be independent of morphological changes.

Actin polymerized as filaments serve as the substrate for the actin-myosin motors [26], which in turn can remodel filament organization [27]. This mechanical interplay thus inextricably interweaves the biological implications of these processes. Our results here were consistent with this notion of interdependence in that modulation of either actin polymerization or actin-myosin interactions had similar effects. We found that, in terms of these cytoskeletal processes, a sufficient level of each was necessary for mesodermal differentiation and a decrease in either promoted endodermal differentiation. Further studies that explore changes in the ratios of the agonists and antagonists would elucidate any compensatory mechanisms that exist between these two cytoskeletal processes. The studies performed here, however, do identify a role of the cytoskeleton in mesendodermal specification, which is required for differentiation towards metabolically active phenotypes, such as hepatocytes and islet cells, as well as mechanoresponsive phenotypes, such as cardiomyocytes, endothelial cells, and osteocytes.

### Applied forces and the cytoskeleton

Elements of the physical microenvironment have long been known to affect stem cell fate (as has been reviewed many times [28,29]). It has been shown that externally applied forces (e.g. tension, compression, and shear) regulate cell fate (e.g. viability, proliferation, and differentiation) in both pluripotent [13,30,31,32] and adult stem cells [4,33,34]. Furthermore, the stiffness of the underlying substrate has also been shown to influence stem cell differentiation [35]. These forces in the external microenvironment are counterbalanced, through direct connections at transmembrane proteins, by the cytoskeleton within the cell [36]. In response to external forces, actin, tubulin, and intermediate filaments can act both as load bearing structures that distribute and transmit forces throughout the cell [37,38,39] as well as participate in force-generating intracellular molecular motors [40].

In our own studies, we see a congruency between the effects of applied external forces and perturbations of the cytoskeleton. We have previously shown that ESC differentiation to mesodermal phenotypes is upregulated when exposed to applied fluid shear stress [13,32]. Cell mechanics studies by others have shown that this type of increased external force can load and reorganize the intermediate filament vimentin [41], as well as actin stress fibers [42]. In a consistent manner, our more recent studies have shown that disruptions in the internal cytoskeletal structure lead to a decrease in mesodermal differentiation. We previously showed that vimentin knockout ESCs have decreased mesodermal differentiation in embryoid bodies compared to wild type cells [19]. Then here we saw that an inhibition of actin polymerization or actin-myosin interactions led to a decrease in mesodermal differentiation. While it is not clear the potential role of parallel or downstream biochemical signaling in these scenarios, together the findings of our studies indicate that mesodermal differentiation is positively correlated with changes in cytoskeletal tension, modulated either as an increase through applied forces or a decrease through intracellular modulation.

The mesodermal lineage includes many mechanoresponsive phenotypes, including endothelial cells, osteocytes, and chondrocytes. These mature phenotypes must appropriately sense and respond to mechanical loading for proper functionality to maintain tissue homeostasis [43]. In the case of osteocytes, there is already some indication that the shear-stress mediated differentiation of mesenchymal stem cells towards this phenotype is modulated by the cytoskeleton [44]. Conversely, there are indications that the absence of cytoskeletal loading may favor differentiation towards non-mechanoresponsive phenotypes, such as adipocytes [1]. It is consistent with this growing understanding of the role of mechanics in differentiation that changes in external forces or cytoskeletal loading during early differentiation would affect cell fate. Therefore, systematic approaches to developing step-wise protocols for directed differentiation of stem cells must address the physical microenvironment during early differentiation. Future studies using defined medium and specific growth factors to direct differentiation towards targeted linages will help to elucidate the role of these mechanisms along specific paths of differentiation.

These studies have shown that modulation of actin filaments and the actin-myosin motor affects germ lineage specification of pluripotent stem cells, increasing our overall understanding of differentiation pathways. Specifically, we have identified two cytoskeletal processes that can be effectively targeted for directing differentiation towards select phenotypes *in vitro* for potential subsequent implantation *in vivo*. Overall, these findings can help develop and optimize protocols for generating cell populations for tissue engineering and regenerative medicine therapies.

## Supporting information

S1 Fig Treatment with agonists or antagonists did not result in increased cell death. Differentiating ESCs cultured on 2D substrates were treated with either JASP, CAL A, CYTO D, or BLEBB and evaluated for viability. Live cells fluoresced green and dead cells fluoresced red. All images were taken at the same magnification and the scale bar represents 400 μm. Treatments did not result in any increases in cell death compared to controls.
(TIF)

S2 Fig Altered actin polymerization or actin-myosin interactions disrupt E-cadherin expression in EBs. Histological sections were stained for E-cadherin (ECAD, red) with a nuclear counterstain (blue) with representative images presented. The scale bar represents 100 μm. Control samples had some visible staining in the interior, as well as a continuous layer of ECAD expression at the periphery. JASP and CYTO D treated samples also expressed ECAD at the exterior edges but very little positive expression was observed in the interior of the EBs. EBs treated with CAL A and BLEBB, however, expressed ECAD within the EB but had discontinuous expression at the periphery.
(TIF)

S3 Fig Antagonists of actin polymerization or actin-myosin interactions did not maintain pluripotency of ESCs. Gene expression of pluripotency markers (*Nanog*, *Oct4*, and *Sox2*) were evaluated in treated samples. Asterisks indicate significance between treatment groups and matched controls (*p<0.05, **p<0.01, ***p<0.001). Treatment with agonists or antagonists did not maintain pluripotency of cells in this differentiation model. All groups expressed similar or further depressed levels of pluripotency markers compared to controls.
(TIF)

## References

[1] McBeath R, Pirone DM, Nelson CM, Bhadriraju K, Chen CS (2004) Cell shape, cytoskeletal tension, and RhoA regulate stem cell lineage commitment. Developmental cell 6: 483–495. 15068789 10.1016/s1534-5807(04)00075-9

[2] Ruiz SA, Chen CS (2008) Emergence of patterned stem cell differentiation within multicellular structures. Stem cells 26: 2921–2927. doi: 10.1634/stemcells.2008-0432 18703661 10.1634/stemcells.2008-0432 PMC2693100

[3] Bao X, Lian X, Hacker TA, Schmuck EG, Qian T, Bhute VJ, et al (2016) Long-term self-renewing human epicardial cells generated from pluripotent stem cells under defined xeno-free conditions. Nature biomedical engineering 1. 10.1038/s41551-016-0003 PMC5408455 28462012

[4] Terraciano V, Hwang N, Moroni L, Park HB, Zhang Z, Mizrahi J, et al (2007) Differential response of adult and embryonic mesenchymal progenitor cells to mechanical compression in hydrogels. Stem cells 25: 2730–2738. doi: 10.1634/stemcells.2007-0228 17702983 10.1634/stemcells.2007-0228

[5] Walker A, Su H, Conti MA, Harb N, Adelstein RS, Sato N (2010) Non-muscle myosin II regulates survival threshold of pluripotent stem cells. Nature communications 1: 71 doi: 10.1038/ncomms1074 20842192 10.1038/ncomms1074 PMC3430968

[6] Chen G, Hou Z, Gulbranson DR, Thomson JA (2010) Actin-myosin contractility is responsible for the reduced viability of dissociated human embryonic stem cells. Cell stem cell 7: 240–248. doi: 10.1016/j.stem.2010.06.017 20682449 10.1016/j.stem.2010.06.017 PMC2916864

[7] Liang G, Zhang Y (2013) Genetic and epigenetic variations in iPSCs: potential causes and implications for application. Cell stem cell 13: 149–159. doi: 10.1016/j.stem.2013.07.001 23910082 10.1016/j.stem.2013.07.001 PMC3760008

[8] Lister R, Pelizzola M, Kida YS, Hawkins RD, Nery JR, Hon G, et al (2011) Hotspots of aberrant epigenomic reprogramming in human induced pluripotent stem cells. Nature 471: 68–73. doi: 10.1038/nature09798 21289626 10.1038/nature09798 PMC3100360

[9] Daniels BR, Hale CM, Khatau SB, Kusuma S, Dobrowsky TM, Gerecht S, et al (2010) Differences in the microrheology of human embryonic stem cells and human induced pluripotent stem cells. Biophysical journal 99: 3563–3570. doi: 10.1016/j.bpj.2010.10.007 21112280 10.1016/j.bpj.2010.10.007 PMC2998615

[10] Boraas LC, Guidry JB, Pineda ET, Ahsan T (2016) Cytoskeletal Expression and Remodeling in Pluripotent Stem Cells. PloS one 11: e0145084 doi: 10.1371/journal.pone.0145084 26771179 10.1371/journal.pone.0145084 PMC4714815

[11] Pineda ET, Nerem RM, Ahsan T (2013) Differentiation patterns of embryonic stem cells in two- versus three-dimensional culture. Cells, tissues, organs 197: 399–410. doi: 10.1159/000346166 23406658 10.1159/000346166 PMC3732738

[12] Keller G (2005) Embryonic stem cell differentiation: emergence of a new era in biology and medicine. Genes & development 19: 1129–1155. 15905405 10.1101/gad.1303605

[13] Wolfe RP, Leleux J, Nerem RM, Ahsan T (2012) Effects of shear stress on germ lineage specification of embryonic stem cells. Integrative biology: quantitative biosciences from nano to macro 4: 1263–1273. 22968330 10.1039/c2ib20040f

[14] Bubb MR, Senderowicz AM, Sausville EA, Duncan KL, Korn ED (1994) Jasplakinolide, a cytotoxic natural product, induces actin polymerization and competitively inhibits the binding of phalloidin to F-actin. The Journal of biological chemistry 269: 14869–14871. 8195116

[15] Goddette DW, Frieden C (1985) The binding of cytochalasin D to monomeric actin. Biochemical and biophysical research communications 128: 1087–1092. 4004848 10.1016/0006-291x(85)91051-4

[16] Ishihara H, Martin BL, Brautigan DL, Karaki H, Ozaki H, Kato Y, et al (1989) Calyculin A and okadaic acid: inhibitors of protein phosphatase activity. Biochemical and biophysical research communications 159: 871–877. 2539153 10.1016/0006-291x(89)92189-x

[17] Kovacs M, Toth J, Hetenyi C, Malnasi-Csizmadia A, Sellers JR (2004) Mechanism of blebbistatin inhibition of myosin II. The Journal of biological chemistry 279: 35557–35563. doi: 10.1074/jbc.M405319200 15205456 10.1074/jbc.M405319200

[18] Heo J, Lee JS, Chu IS, Takahama Y, Thorgeirsson SS (2005) Spontaneous differentiation of mouse embryonic stem cells in vitro: characterization by global gene expression profiles. Biochemical and biophysical research communications 332: 1061–1069. doi: 10.1016/j.bbrc.2005.04.173 15922302 10.1016/j.bbrc.2005.04.173

[19] Boraas LC, Ahsan T (2016) Lack of vimentin impairs endothelial differentiation of embryonic stem cells. Scientific reports 6: 30814 doi: 10.1038/srep30814 27480130 10.1038/srep30814 PMC4969593

[20] Feng T, Szabo E, Dziak E, Opas M (2010) Cytoskeletal disassembly and cell rounding promotes adipogenesis from ES cells. Stem cell reviews 6: 74–85. doi: 10.1007/s12015-010-9115-8 20148318 10.1007/s12015-010-9115-8

[21] Cai Y, Rossier O, Gauthier NC, Biais N, Fardin MA, Zhang X, et al (2010) Cytoskeletal coherence requires myosin-IIA contractility. Journal of cell science 123: 413–423. doi: 10.1242/jcs.058297 20067993 10.1242/jcs.058297 PMC2816186

[22] Fletcher DA, Mullins RD (2010) Cell mechanics and the cytoskeleton. Nature 463: 485–492. doi: 10.1038/nature08908 20110992 10.1038/nature08908 PMC2851742

[23] Thomas CH, Collier JH, Sfeir CS, Healy KE (2002) Engineering gene expression and protein synthesis by modulation of nuclear shape. Proceedings of the National Academy of Sciences of the United States of America 99: 1972–1977. doi: 10.1073/pnas.032668799 11842191 10.1073/pnas.032668799 PMC122304

[24] Watt FM, Jordan PW, O'Neill CH (1988) Cell shape controls terminal differentiation of human epidermal keratinocytes. Proceedings of the National Academy of Sciences of the United States of America 85: 5576–5580. 2456572 10.1073/pnas.85.15.5576 PMC281801

[25] Kilian KA, Bugarija B, Lahn BT, Mrksich M (2010) Geometric cues for directing the differentiation of mesenchymal stem cells. Proceedings of the National Academy of Sciences of the United States of America 107: 4872–4877. doi: 10.1073/pnas.0903269107 20194780 10.1073/pnas.0903269107 PMC2841932

[26] Cooper GM (2000) The Cell—A Molecular Approach 2nd Edition.

[27] Vicente-Manzanares M, Ma X, Adelstein RS, Horwitz AR (2009) Non-muscle myosin II takes centre stage in cell adhesion and migration. Nature reviews Molecular cell biology 10: 778–790. doi: 10.1038/nrm2786 19851336 10.1038/nrm2786 PMC2834236

[28] Han YL, Wang S, Zhang X, Li Y, Huang G, Qi H, et al (2014) Engineering physical microenvironment for stem cell based regenerative medicine. Drug discovery today 19: 763–773. doi: 10.1016/j.drudis.2014.01.015 24508818 10.1016/j.drudis.2014.01.015

[29] Sun Y, Chen CS, Fu J (2012) Forcing stem cells to behave: a biophysical perspective of the cellular microenvironment. Annual review of biophysics 41: 519–542. doi: 10.1146/annurev-biophys-042910-155306 22404680 10.1146/annurev-biophys-042910-155306 PMC4123632

[30] McKee C, Hong Y, Yao D, Chaudhry GR (2017) Compression induced chondrogenic differentiation of embryonic stem cells in 3-D PDMS scaffolds. Tissue engineering Part A. 10.1089/ten.TEA.2016.0376 28103756

[31] Teramura T, Takehara T, Onodera Y, Nakagawa K, Hamanishi C, Fukuda K (2012) Mechanical stimulation of cyclic tensile strain induces reduction of pluripotent related gene expressions via activation of Rho/ROCK and subsequent decreasing of AKT phosphorylation in human induced pluripotent stem cells. Biochemical and biophysical research communications 417: 836–841. doi: 10.1016/j.bbrc.2011.12.052 22206673 10.1016/j.bbrc.2011.12.052

[32] Wolfe RP, Ahsan T (2013) Shear stress during early embryonic stem cell differentiation promotes hematopoietic and endothelial phenotypes. Biotechnology and bioengineering 110: 1231–1242. doi: 10.1002/bit.24782 23138937 10.1002/bit.24782 PMC4052571

[33] Luo W, Xiong W, Zhou J, Fang Z, Chen W, Fan Y, et al (2011) Laminar shear stress delivers cell cycle arrest and anti-apoptosis to mesenchymal stem cells. Acta biochimica et biophysica Sinica 43: 210–216. doi: 10.1093/abbs/gmr004 21335336 10.1093/abbs/gmr004

[34] Kurpinski K, Chu J, Hashi C, Li S (2006) Anisotropic mechanosensing by mesenchymal stem cells. Proceedings of the National Academy of Sciences of the United States of America 103: 16095–16100. doi: 10.1073/pnas.0604182103 17060641 10.1073/pnas.0604182103 PMC1637542

[35] Engler AJ, Sen S, Sweeney HL, Discher DE (2006) Matrix elasticity directs stem cell lineage specification. Cell 126: 677–689. doi: 10.1016/j.cell.2006.06.044 16923388 10.1016/j.cell.2006.06.044

[36] Ingber DE (1993) Cellular tensegrity: defining new rules of biological design that govern the cytoskeleton. Journal of cell science 104 (Pt 3): 613–627. 8314865 10.1242/jcs.104.3.613

[37] Pourati J, Maniotis A, Spiegel D, Schaffer JL, Butler JP, Fredberg JJ, et al (1998) Is cytoskeletal tension a major determinant of cell deformability in adherent endothelial cells? The American journal of physiology 274: C1283–1289. 9612215 10.1152/ajpcell.1998.274.5.C1283

[38] Wang N, Butler JP, Ingber DE (1993) Mechanotransduction across the cell surface and through the cytoskeleton. Science 260: 1124–1127. 7684161 10.1126/science.7684161

[39] Buxboim A, Ivanovska IL, Discher DE (2010) Matrix elasticity, cytoskeletal forces and physics of the nucleus: how deeply do cells 'feel' outside and in? Journal of cell science 123: 297–308. doi: 10.1242/jcs.041186 20130138 10.1242/jcs.041186 PMC2816180

[40] Discher DE, Janmey P, Wang YL (2005) Tissue cells feel and respond to the stiffness of their substrate. Science 310: 1139–1143. doi: 10.1126/science.1116995 16293750 10.1126/science.1116995

[41] Helmke BP, Goldman RD, Davies PF (2000) Rapid displacement of vimentin intermediate filaments in living endothelial cells exposed to flow. Circulation research 86: 745–752. 10764407 10.1161/01.res.86.7.745

[42] Franke RP, Grafe M, Schnittler H, Seiffge D, Mittermayer C, Drenckhahn D (1984) Induction of human vascular endothelial stress fibres by fluid shear stress. Nature 307: 648–649. 6537993 10.1038/307648a0

[43] Kamkin A, Kiseleva I (2005) Mechanosensitivity of Cells from Various Tissues. In: Kamkin A, Kiseleva I, editors. Mechanosensitivity in Cells and Tissues. Moscow.

[44] Arnsdorf EJ, Tummala P, Kwon RY, Jacobs CR (2009) Mechanically induced osteogenic differentiation—the role of RhoA, ROCKII and cytoskeletal dynamics. Journal of cell science 122: 546–553. doi: 10.1242/jcs.036293 19174467 10.1242/jcs.036293 PMC2714434

