# Supplementary figures and images for: Actin and myosin II modulate differentiation of pluripotent stem cells
Source: PLoS One. 2018 Apr 17;13(4):e0195588. doi: 10.1371/journal.pone.0195588 (PMC5903644; doi:10.1371/journal.pone.0195588)

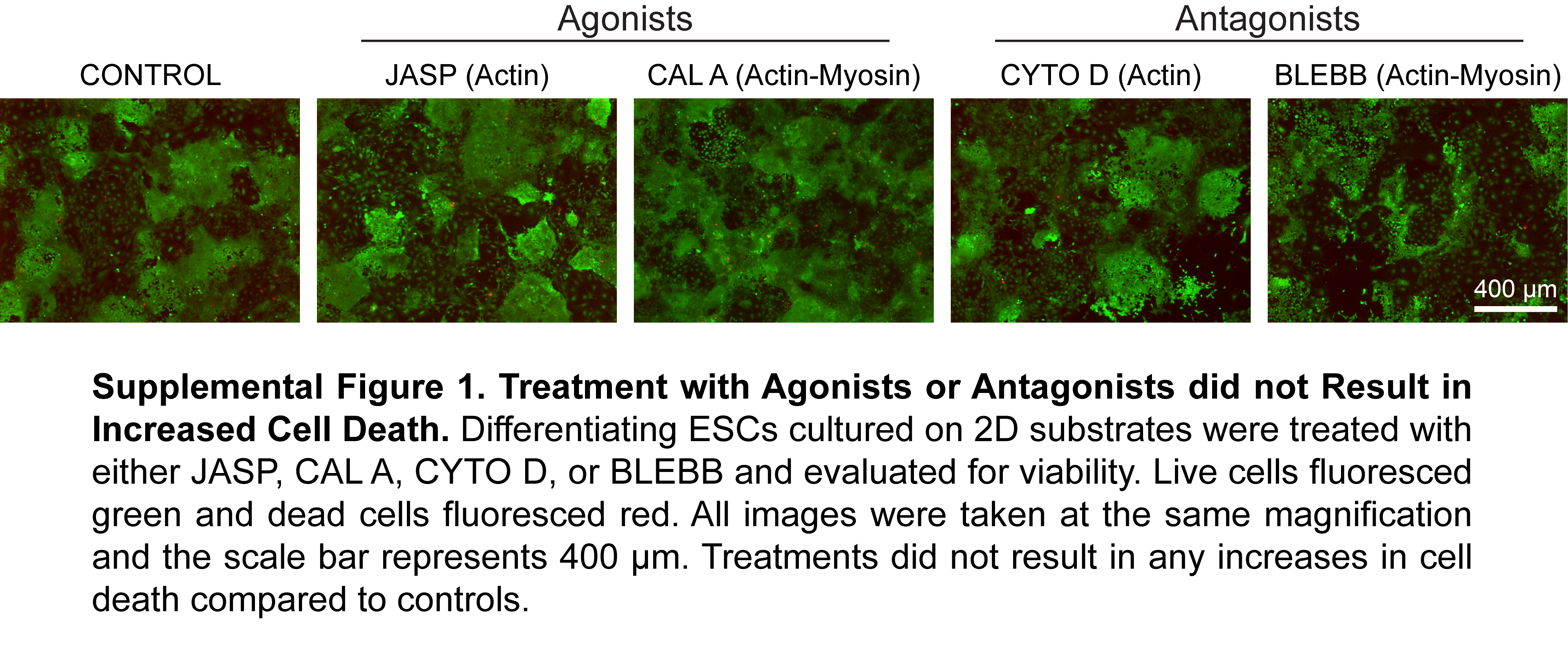

Supplement: S1 Fig — Differentiating ESCs cultured on 2D substrates were treated with either JASP, CAL A, CYTO D, or BLEBB and evaluated for viability. Live cells fluoresced green and dead cells fluoresced red. All images were taken at the same magnification and the scale bar represents 400 μm. Treatments did not result in any increases in cell death compared to controls. (TIF) [file pone.0195588.s001.tif]

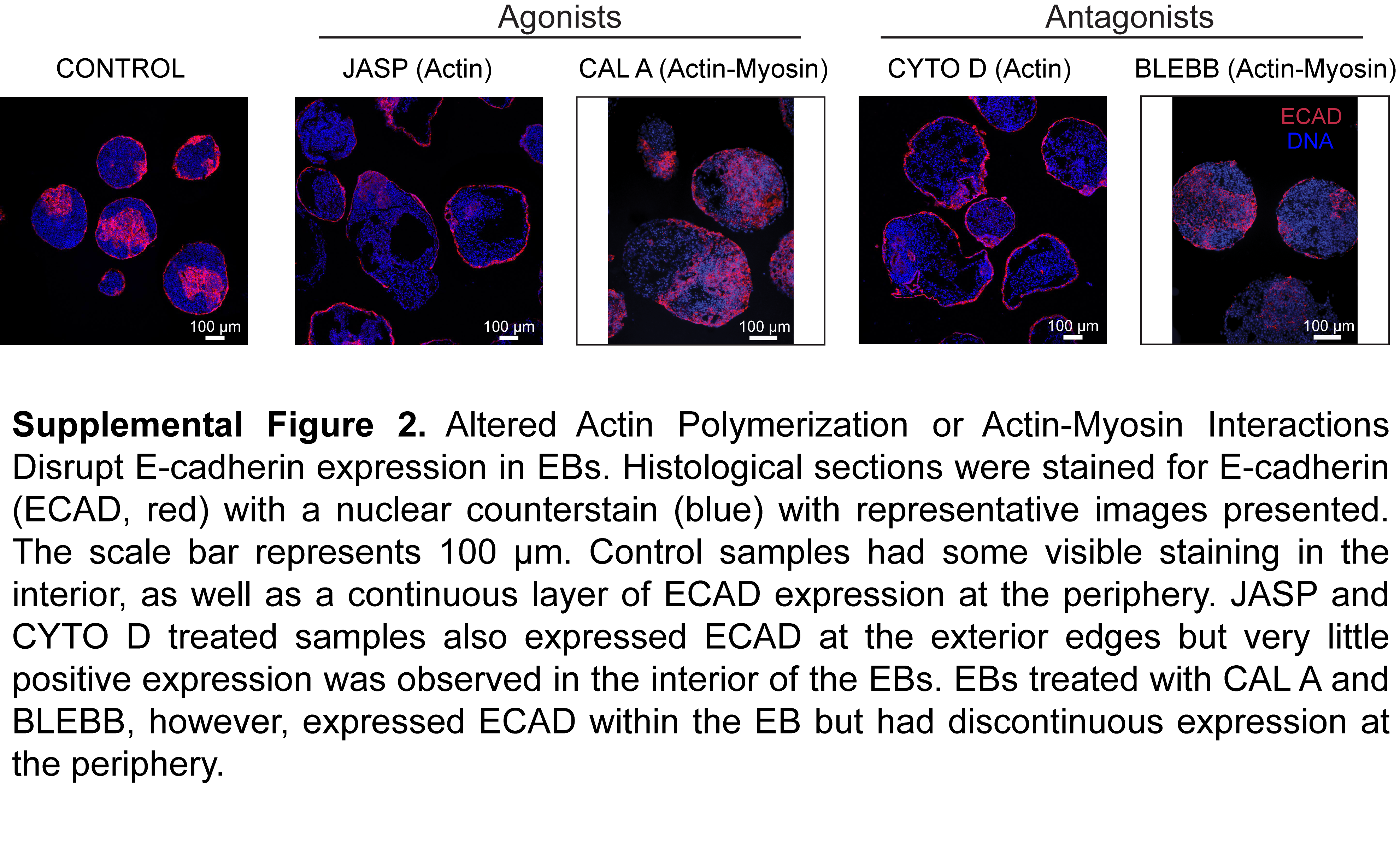

Supplement: S2 Fig — Histological sections were stained for E-cadherin (ECAD, red) with a nuclear counterstain (blue) with representative images presented. The scale bar represents 100 μm. Control samples had some visible staining in the interior, as well as a continuous layer of ECAD expression at the periphery. JASP and CYTO D treated samples also expressed ECAD at the exterior edges but very little positive expression was observed in the interior of the EBs. EBs treated with CAL A and BLEBB, however, expressed ECAD within the EB but had discontinuous expression at the periphery. (TIF) [file pone.0195588.s002.tif]

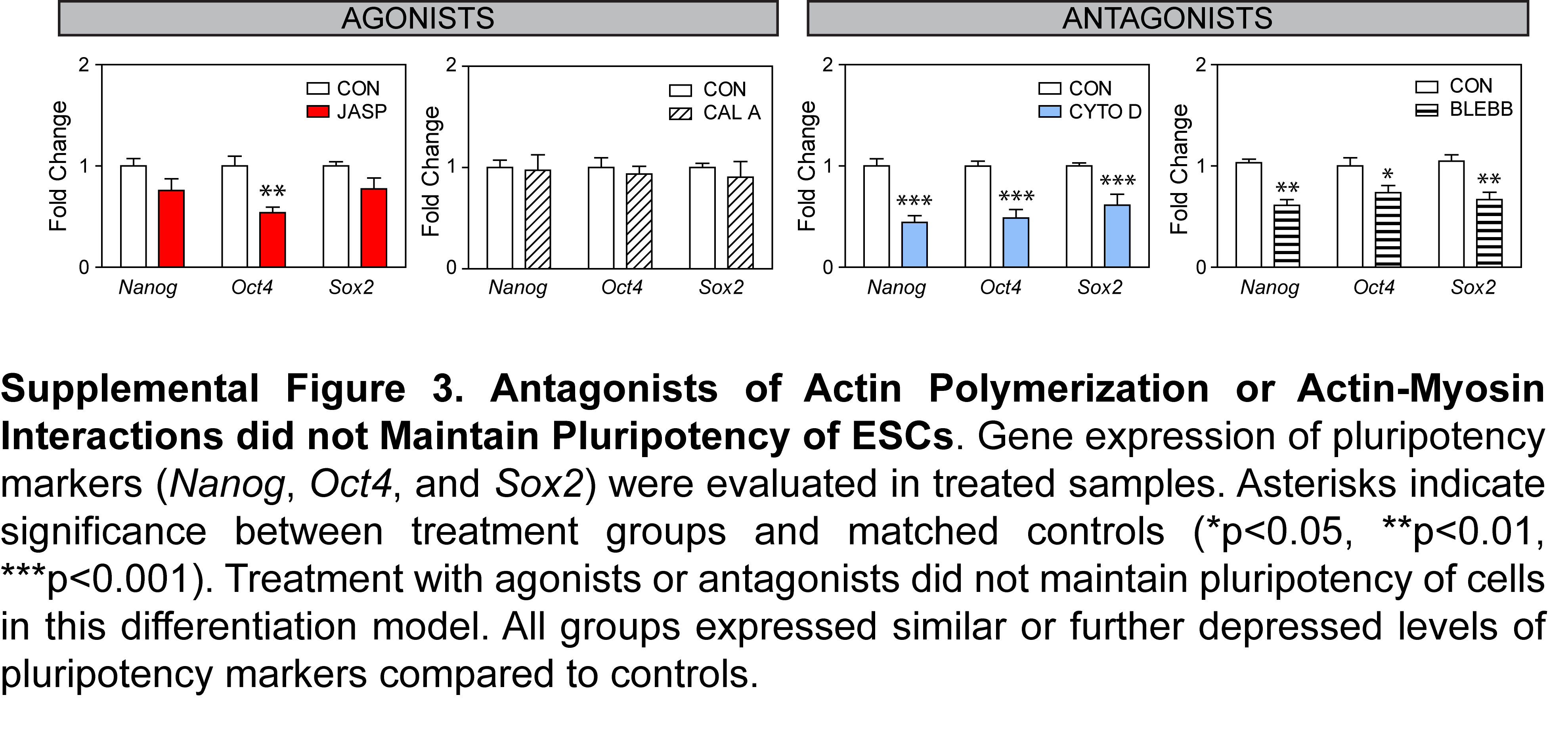

Supplement: S3 Fig — Gene expression of pluripotency markers (Nanog, Oct4, and Sox2) were evaluated in treated samples. Asterisks indicate significance between treatment groups and matched controls (*p<0.05, **p<0.01, ***p<0.001). Treatment with agonists or antagonists did not maintain pluripotency of cells in this differentiation model. All groups expressed similar or further depressed levels of pluripotency markers compared to controls. (TIF) [file pone.0195588.s003.tif]
